# Challenges to Implementing a Vaccine for Coccidioidomycosis

**DOI:** 10.1093/ofid/ofae095

**Published:** 2024-02-19

**Authors:** Bridget M Barker, George R Thompson, Neil M Ampel

**Affiliations:** Department of Biological Sciences, Northern Arizona University, Flagstaff, Arizona, USA; Department of Medical Microbiology and Immunology, University of California at Davis, Davis, California, USA; Departments of Medicine and Immunobiology, University of Arizona, Tucson, Arizona, USA

**Keywords:** coccidioidomycosis, immunization, mycoses, vaccination

## Abstract

A vaccine for coccidioidomycosis is likely to undergo trials in the near future. In this paper, we raise 4 questions that should be answered before its use and offer our solutions to these questions. These include defining the goals of vaccination, determining who should be vaccinated, how to measure vaccine immunity and protection, and how to address vaccine hesitancy and denial.

Fungal infections are an emerging global problem [1]. This has led to proposals for newer approaches for their control and treatment. One is the development of vaccines. Until recently, no fungal vaccine has been available. However, the promising results of a recent canine study using a live attenuated vaccine (Δ*cps1*) have been published [2]. In addition to this product, several other coccidioidal vaccines are in various stages of development, including those based on genomic technologies and those using purified peptide and protein subunits [3, 4]. Because of this, we are likely to see studies of a human coccidioidal vaccine in the near future. Before implementing any such vaccine studies, we believe there are several challenges that need to be addressed. In this paper, we discuss the coccidioidal life cycle and immune response and propose four questions to be considered during the development of a vaccine and propose our answers to these questions. We believe these questions are independent of the vaccine platform and discussion of specific immunization approaches in this paper will be limited.

## COCCIDIOIDAL LIFE CYCLE AND IMMUNITY

Fungi are not frequent human pathogens. Among those that are associated with infection, except for *Candida* and possibly *Pneumocystis* species, all exist in the environment and do not require a human host for survival. In the case of coccidioidomycosis, the precise environmental niche has not been established, but the fungus has been documented to persist in soils within as well as outside the expected endemic range [5]. Once established in particular sites, the fungus may remain there for many years [6, 7]. In addition, the fungus has been found in the air over large geographic areas for prolonged periods within the endemic region [8, 9]. These data suggest that *Coccidioides* is extant in both the soil and air environment within its geographic niche.

Most coccidioidal infection occurs from inhalation of infectious airborne spores called arthroconidia. Currently, it is presumed that annual risk of infection within the endemic region is between 0.5% and 1.6% [10, 11] and that from 20% to 43% of those living in the coccidioidal endemic regions are infected [12–14]. However, these studies are broad estimates that have not been recently updated. Moreover, the risk of acquiring coccidioidal infection within any particular endemic region is likely strongly influenced by local soil and climate conditions and depends on undefined stochastic events. Because of this, we cannot currently predict individual risk for acquiring coccidioidal infection among those living in the endemic regions and we are unlikely to be able to completely mitigate this risk.

For *Coccidioides* and other dimorphic fungi, the host–pathogen interaction is extremely complex. Our understanding of this interaction has been informed by recent insights from Taylor and Barker [15], who proposed a new model of endozoan coccidioidal infection in which infection of small mammalian hosts is inherent to the life cycle of the fungus, leading to a persistent reservoir. This model also applies to human infection where, in most individuals, coccidioidal infection is persistent but latent. Smith and colleagues [16] demonstrated in a prospective study many decades ago that 60% of those infected do not come to clinical attention and have stable long-lived immunity associated with development of a delayed dermal hypersensitivity reaction, a hallmark of cellular immunity. Among the 40% with symptoms, most have a self-limited respiratory infection and many never seek medical care. A small fraction either develop pulmonary sequelae from infection [17] or manifest infection outside the thoracic cavity, called dissemination [18]. Overall, only about 5% of those infected with *Coccidioides* require long-term management [19]. Clinically apparent second infections are extremely rare [20] and recrudescence of infection, once controlled, is infrequent. However, the fungus remains viable in the host for many years [21], possibly for life.

Certain groups of individuals are at risk for either severe pulmonary or disseminated infection. These include those with suppressed cellular immunity, such as untreated HIV-1 infection with immunodeficiency [22], solid organ transplant recipients [23], and those on immunosuppressive drugs, such as certain biological response modifiers [24] and corticosteroids [25]. In addition, individuals with an African or Oceanic genetic ancestry [26, 27], particularly men [28], and pregnant women who acquire infection during and after the second trimester [29] are at increased risk for severe disease.

## FOUR QUESTIONS FOR THE DEVELOPMENT OF A COCCIDIOIDAL VACCINE

### What Are the Goals of Vaccination?

Based on the fungal life cycle and host immune response, it is unlikely that a successful vaccine will prevent coccidioidal infection. Instead, it is probable that it will result in enhanced control of subsequent infection through a vaccine-induced specific cellular immune response. The primary goal of vaccination would thus be to prevent symptomatic primary pneumonia. If such a vaccine were effective, it would reduce initial visits to primary and urgent care clinics and emergency rooms as well as reduce unnecessary antibiotic prescriptions, repeat clinic visits, and lead to an overall reduction in the documented health care costs [30]. Based on earlier data, development of a coccidioidal vaccine has been proposed to be cost-effective [31].

However, that cost is likely to be substantial. A recent estimate of the for developing a new vaccine through a phase 2a trial for an epidemic infectious disease in the United States was $2.8 to 3.7 billion [32]. In addition, as Kirkland has pointed out, a reliable pharmaceutical partner will have to be found [33], a difficulty already noted for studies of new antifungal therapeutic agents for coccidioidomycosis [34]. Because of this, bringing a new coccidioidal vaccine through trials and to market will be financially daunting. On the other hand, Galgiani and colleagues have addressed these issues and believe they are surmountable through a public–private consortium at a cost of $200 to $300 million [35].

Another goal could be to reduce the risk of extrathoracic dissemination. Observational studies suggest that this occurs in approximately 1% of those infected [16] but may be higher in those with symptomatic illness [36]. It is usually associated with a diminished expression of cellular immunity [37, 38]. Prevention of this manifestation would be very desirable because these patients have significant morbidity, occasional mortality, and require prolonged clinical follow-up and therapy with antifungals. These patients have substantial lifetime costs of more than $1 million per patient [39], so an effective vaccine would lead to financial as well as health benefits. However, it is unknown at this time if a vaccine will induce appropriate protective immunity in those at risk for dissemination because these patients appear to have a lack of response to natural infection. Because of this, specific studies to assess vaccine efficacy in this group will be needed and will likely require a larger number of subjects [33] and longer term follow-up than for primary infection. We estimate, based on an annual incidence of coccidioidal infection of 1.5% with a 1% dissemination rate and assuming that all disseminations will occur within 2 years of infection, approximately 5000 subjects would have to be followed for at least 7 years to ascertain if a vaccine reduces this risk. Because of the difficulty and costs of designing such a trial, we believe that prevention of extrathoracic dissemination need not be a primary goal of development of a coccidioidal vaccine and could be addressed after such a vaccine has reached the market.

### Who Should be Vaccinated?

An obvious target would be all persons who are at risk for coccidioidal infection. Although the highly endemic coccidioidal regions are well described and include the southern portion of the San Joaquin Valley and south-central Arizona [40], coccidioidomycosis may be acquired in many other areas that are not as well demarcated [41]. In addition, should those at higher risk because of exposure, such as outdoor workers [42], be a priority? Should those visiting the endemic area for vacation and recreation [43] be considered? What duration of exposure to the endemic area would necessitate vaccination? Although this issue could wait until there is an effective vaccine, it is reasonable to consider these questions now for both research and marketing purposes.

We propose that all individuals living within known highly endemic regions, particularly the San Joaquin Valley and central Arizona encompassing Maricopa, Pinal, and Pima Counties, be considered for vaccination. Persons considered at higher risk based on exposure, such as military personnel training in areas of known endemicity [44], outdoor workers, especially those employed in agriculture [45], utilities [46], and wildland firefighters [47], and prisoners and prison workers at facilities located within these known highly endemic areas [48] should be populations of particular focus.

It is not clear if those with prior coccidioidal infection would benefit from immunization, having already acquired infection and protective immunity. Should those with prior infection be screened and excluded? We believe that for initial studies to ascertain vaccine efficacy, individuals with prior infection should not be included [14, 33]. Otherwise, the efficacy of the vaccine might be significantly overestimated. However, to achieve this, tools to identify such individuals will need to be available.

An important area of study is immunization of those who have known depressed cellular immunity or other risks for severe coccidioidomycosis based on possible genetic polymorphisms [49]. Prior immunization should be considered in cases in which immunosuppression is planned and is iatrogenic, such as in solid organ transplant candidates and those being considered for biological response modifier therapy. It also would be reasonable for women considering pregnancy to be given the vaccine before conceiving. Whether immunization will be effective after immunosuppression has occurred or for those with an underlying immune deficit is unclear and should be an area of study. Mice with various types of cellular immunodeficiency were able to control coccidioidal infection after vaccination with the Δ*cps1* live attenuated knockout [50]. Other vaccines have induced protection in mice highly susceptible to coccidioidal infection [33]. However, animal models of vaccine efficacy may not predict efficacy in humans [51], and human trials will be required. Initial trials to determine vaccine efficacy need not include these individuals and use of vaccine among these groups of patients should be considered once the vaccine is available. Postmarket data collection in these cases will be critical.

### How Should Vaccine Effectiveness and Prior Infection be Measured?

A definitive way to measure vaccine effectiveness is to determine the number of active coccidioidomycosis cases that occur in those vaccinated compared to those not immunized. However, that depends on the incidence of coccidioidomycosis during the study period, the number of subjects entered, as well as the endpoints and duration of follow-up. A prior study of a coccidioidal vaccine, made from formaldehyde-killed spherules, is instructive [14]. The vaccine used was in a lower concentration than that found effective in mice [52] because of unfavorable injection-site reactions in human subjects [53]. The trial was conducted between 1980 and 1985 and included 1436 subjects who received 3 intramuscular injections of vaccine and 1431 who received placebo injections over a 6-week period. All subjects were coccidioidal skin-test negative on study entry. It was anticipated that 68 cases of coccidioidomycosis would occur in the placebo arm. However, after an average follow-up of 2.3 years, 9 cases of coccidioidomycosis were noted in vaccine recipients compared with 12 cases in those receiving placebo, not a statistically significant difference and well below the number of cases anticipated. None of the instances of coccidioidomycosis that occurred was disseminated. Overall, the trial was not considered successful [54] and further studies of formaldehyde-killed spherules as a vaccine were not pursued. Any future study of vaccine efficacy should use these same endpoints but ensure adequate subject recruitment to determine vaccine efficacy.

An indirect mechanism for determining vaccine efficacy by measuring the expression of coccidioidal-specific cellular immune response could be useful. Smith and colleagues showed the expression of delayed-type dermal hypersensitivity after a skin test predicted a good outcome and control of infection in most cases, with the exception of coccidioidal meningitis [37, 55]. Oldfield and colleagues have suggested that development of skin-test positivity is associated with a diminished risk of relapse after completion of antifungal therapy [56] and delayed-type hypersensitivity occurred predictably in a healthy cohort with recent primary pulmonary coccidioidomycosis using a reformulated spherulin-based product [57]. However, 2 recent reports examining coccidioidal skin-testing were not necessarily predictive of immunity [58, 59], and the skin test has not always detected immunity in patients with known prior coccidioidomycosis [60]. Based on this, the role of coccidioidal skin testing as a prognostic tool is not established. We do not advocate using the skin test in future studies for determining coccidioidal immunity.

A more modern approach is to measure ex vivo T-cell activation. Older methods used lymphocyte transformation [61, 62], but measurement of cytokine release or expression by either whole blood or blood cellular components has more recently been explored [38, 63–67]. A correlation with clinical expression of control of disease has been noted [38, 65]. However, this issue has not been subjected to a rigorous prospective study. If surrogate endpoints for immunity are contemplated, we strongly advocate for studying these assays to ascertain their prognostic usefulness and such studies should be an early part of any coccidioidal vaccine strategy.

In addition, determining the duration of vaccine immunity will be critical. Were vaccine immunity to fade, those remaining within the endemic region could again become susceptible to infection and illness. If the model that protective immunity persists in coccidioidomycosis because of the persistence of live fungal elements, then a vaccine would have to induce that state. Of the current vaccine candidates, only a live attenuated vaccine could potentially achieve this goal, but that has not been established in humans. The other possible candidates, based on nucleic acid technology or subunit vectors, would not lead to antigen persistence. This could mean that their duration might be short lived. However, recent advances in vaccine adjuvants suggests that long-lived cellular immunity can occur with subunit vaccines [68, 69] and mRNA vaccines can act as self-adjuvants [70], potentially abrogating this problem. Because of this, we recommend long-term postvaccine studies to ascertain the persistence of protective immunity either by indirect means, such as cytokine release assays, or by determining if new clinical infections occur after vaccination.

### How do we Approach Vaccine Hesitancy?

Vaccine hesitancy, a delay or refusal to be vaccinated [71], dates back to the first smallpox and cowpox immunizations in the 18th century [72]. It is a global issue that is heterogeneous, individualized, and exacerbated since the development of the internet and social media [71–73]. We should anticipate that there will be some level of hesitancy with the availability of a coccidioidal vaccine. The vaccine will be novel and the first directed at a fungal infection. At this time, it appears that it will either use a gene knock-out strain of *Coccidioides,* a nucleic acid platform, or be composed of subunit peptides or proteins with an adjuvant [74, 75]. These approaches are very likely to engender concern among some members of the public.

How quickly vaccine hesitancy can occur is demonstrated by events associated with the development of a vaccine for Lyme disease [76]. In 1998, the US Food and Drug Administration licensed LYMErix, a vaccine targeting the outer surface protein A of *Borrelia burgdorferi*. Approval was based on a phase III trial demonstrating 76% efficacy and only mild to moderate short-term local or systemic adverse events [77]. However, reports of arthritis appeared after soon after licensure. These were associated with a class action suit and concern expressed by a Lyme disease advocacy group. The Food and Drug Administration subsequently examined data from the Vaccine Adverse Events Reporting System and did not find an excess of arthritis episodes in those receiving the vaccine. However, the possibility of an outer surface protein A autoimmunity event associated with an HLA DR allele was raised. The vaccine was ultimately voluntarily withdrawn by the manufacturer in 2002 because of poor market performance [76].

The COVID-19 epidemic has been associated with a marked increase in both vaccine hesitancy and denial [78] and is likely to increase the headwinds toward patient acceptance of a coccidioidal vaccine. With regard to COVID-19, vaccine hesitancy not only involved individual concerns about the efficacy and safety of the specific vaccines but also mistrust of government, science, and vaccines in general. This was exacerbated by social media platforms willing to spread vaccine-related misinformation [73]. Such issues will need to be addressed prior to marketing a coccidioidal vaccine.

Although it is possible that the public attitude toward a coccidioidal vaccine will be different than for other vaccines, this cannot be assumed. A novel coccidioidal vaccine will require a specific plan to educate the public about its benefits and potential risks. This will require a multipronged approach directed at education from trusted sources, examination of social and cultural values, and retooling of health-related media platforms to combat misinformation [79]. In addition, the populations initially targeted for vaccination should be those who are most at risk and would most benefit from vaccination. Long-term follow-up must be inherent to the vaccine strategy to ensure that no late effects occur that are not anticipated and to openly report data from follow-ups.

We propose that funding agencies make vaccine uptake, hesitancy, and denial a part of their strategy to develop a coccidioidal vaccine now. It would be prudent to involve public coccidioidomycosis advocacy groups in this process so they can express their concerns early so that they can be addressed prior to a product coming to market.

***Patient Consent Statement.*** No human subjects were involved in the preparation of this manuscript.

***Financial support*.** No funding was used in the preparation of this manuscript.
